# The factors in older adults’ health literacy in the field of physical activity: a qualitative study

**DOI:** 10.1186/s12877-022-03320-z

**Published:** 2022-07-30

**Authors:** Moeini Babak, Barati Majid, Heidarimoghadam Rashid, Tapak Leili, Parsamajd Shahryar

**Affiliations:** 1 Social Determinants of Health Research Center and Department of Public Health, School of Public Health, Hamadan University of Medical Sciences, Hamadan, Iran; 2 Research Center for Health Sciences and Department of Ergonomics, School of Public Health, Hamadan University of Medical Sciences, Hamadan, Iran; 3 Department of Biostatistics, School of Public Health, Modeling of Noncommunicable Diseases Research Center, Hamadan University of Medical Sciences, Hamadan, Iran; 4 Department of Public Health, School of Public Health and Student Research Committee, Health Faculty, Hamadan University of Medical Science, Shahid Fahmide Boulevard, Hamadan, 8138380509 Iran

**Keywords:** Older adults, Health literacy, Physical activity

## Abstract

**Background:**

Older adults are one of the most vulnerable groups to the undesirable effects of low health literacy. Inadequate health literacy in older adults is associated with decreased physical activity, deviation from the path of health, and suffering from various diseases. Considering the role and importance of health literacy in promoting physical activity and improving health in older adults and the hypothesis that there are certain factors associated with health literacy in the field of physical activity, this study is aimed at understanding the factors related to older adults’ health literacy about physical activity.

**Methods:**

This study is a qualitative study on older adults 60 to 75 years old in retirement centers in Kermanshah, Iran, in 2020. Totally, 25 participants were recruited through purposeful sampling with maximum variation until data saturation. The data were collected through in-depth semi-structured interviews and analyzed using directed qualitative content analysis.

**Results:**

By analyzing the manuscripts obtained from the interviews, 59 initial codes were extracted, which were reduced to 32 main codes after careful assessment. The main codes were grouped into 13 subcategories under 5 categories. Subcategories are the detected factors that are related to health literacy and categories are the five aspects of health literacy including access, reading skill, comprehension, evaluation and decision-making, and application of information. Health literacy was the main theme that encompassed the categories.

**Conclusion:**

This study provided a comprehensive understanding of beliefs, opinions and factors related to older adults’ health literacy about physical activity. According to these findings, physical problems and diseases are not obstacles to making decisions and applying physical activity information in older adults who have a high understanding and proper evaluation of physical activity recommendations. Support, advertising, and organizational facilities are related to all dimensions of older adults’ health literacy about physical activity, while socio-economic factors are related to the dimensions of access, comprehension, decision-making, and application of information. The factors related to older adults’ health literacy about physical activity that were identified in this study, can be used by organizations that are responsible for policy-making, decision-making, and implementing physical activity promotion programs to improve the health in older adults.

## Background

Like most parts of the world, improved health, treatment, economic, and social indicators have increased life expectancy in Iran and created a bigger older adults population [1]. The main challenge of health in the twentieth century was the mere survival; however, the main concern in the twenty-first century is to live with a superior quality. Being physically active and improvement in quality of life is important in old age as well as the increase in life expectancy and lifespan [2]. Ageing is a complex process of physiological and social developments and physical activity is one of the important strategies in the process of healthy ageing [3].

Regular physical activity reduces the risk of cardiovascular and metabolic diseases, obesity, cognitive disorders, osteoporosis, and muscle weakness [4]. Physical activity helps older adults to be independent and active, suffer less depression and anxiety, improve physical, mental and psychological functions, reduce recurrent falls injuries, sleep well, improve their balance, and maintain muscle strength and endurance [5]. Despite such health benefits, low physical activity is very common in older adults [6].

Health literacy is an important element in the ability of individuals to participate in health-related activities, disease prevention, and treatment decisions [7]. Health literacy affects the motivation to engage in physical activity and exercise [8]. In the view of the world health organization (WHO), health literacy has been introduced as a social cognitive skill that determines the motivation and ability of individuals to achieve, understand and use information in a manner that will lead to the maintenance and promotion of their health. WHO presented health literacy as one of the greatest determinants of health [9]. Health literacy is the capacity to acquire, process, and understand the basic information and services needed to make appropriate health decisions and actions [10]. Limitation of health literacy in vulnerable groups, including older adults, put them at the risk of poor health [11]. Older people are at the risk of adverse effects of low levels of health literacy, which is a warning to health care providers. Health literacy can be effective in controlling and self-managing geriatric illnesses, reducing the burden of health care, and reducing health system costs [12]. Health literacy is related to mortality in the elderly [13]. such that a 5-year follow-up study has reported that the odds of the elderly with low health literacy is twice greater than those of the elderly with high health literacy [14].

Health literacy along with self-efficacy are cognitive factors that have a significant relationship with physical activity in older adults. Inadequate health literacy in older adults is associated with failure to observe physical activity guidelines. Self-efficacy acts as a social cognitive mediator between health literacy and following physical activity guidelines [15]. Aging brings about some cognitive and perceptual problems in the elderly that are obstacles to obtaining information and promoting health literacy [16]. The results of studies have shown that health literacy in the elderly is at a low level [17, 18]. In general, studies on health literacy assessment and its determinants have reported low levels of health literacy in the Iranian older adults and stressed the need to promote health literacy in them [19, 20]. According to a systematic review and meta-analysis, the health literacy of Iranian older adults is below average (45.8 out of 100), [21]. Paying attention to health literacy is essential for promoting health in older adults [22]. By planning and designing useful educational interventions in the field of health literacy in older adults, it is possible to promote their physical health [12]. Improving health literacy in older adults is a new strategy to promote their participation in physical activity [23].

Numerous studies have evaluated the inadequacy of health literacy as one of the factors in low level of the physical activity in older adults [12, 15]. Studies have also examined the factors affecting the general concept of health literacy in older adults [15, 19, 20]. However, no qualitative study has been conducted specifically to examine and explain the factors related to the dimensions of health literacy in older adults (e.g. access, reading skills, comprehension, evaluation, decision-making, and application of information) [11].

The review of scientific literature and evidence has indicated the significance and role of health literacy in maintaining and promoting the health status of the elderly. On the other hand, doing regular physical activities in the elderly plays a role in improving the health and life quality in the elderly. Considering that both health literacy in the broad sense, and regular physical activities have some role in improving health in the elderly, the specific concept of health literacy and physical activities in the elderly has attracted our attention. According to the extensive review of literature thus far we have found no study conducted to examine and detect the specific concept of health literacy and physical activities in the elderly. Therefore, the examination and detection of this concept of innovation show the significance of our study. Our study hypothesis maintains that there are some factors related to the specific concept of health literacy in the elderly and according to this hypothesis, we aimed at understanding these factors. We can develop a specific questionnaire to assess this concept by recognizing the factors relating to the specific concept of health literacy and physical activities of the elderly and use the questionnaire in future intervention studies to plan and promote physical activities and improve the health status of the elderly.

In this study, we have taken into consideration all dimensions of health literacy in the broad sense in order to achieve comprehensive information on the factors related to the specific concept of health literacy and physical activities. According to the five dimensions of health literacy, for instance, if an elderly person intends to do regular physical activities, they should first of all have correct information on physical activities from credible resources. The next stage is the ability to read written media and comprehend received information. Finally, the elderly decide about doing regular physical activities after carrying out correct evaluation and analysis.

Considering the role and importance of health literacy in promoting physical activity and improving health in older adults, this qualitative study, which is part of a theory-based intervention study to promote physical activity in older adults, was conducted to understand the factors related to different dimensions of health literacy in physical activity. Deep knowledge, understanding, and extracting the beliefs of older adults in this field can guide and facilitate the development and implementation of policies and programs to improve physical activity and health in older adults.

## Methods

This study is aimed at understanding the factors related to older adults’ health literacy about physical activity. We conducted a qualitative approach using in-depth semi-structured interviews on older adults in retirement centers in Kermanshah, Iran, in 2020.

### Participants and settings

The participants were selected among older people aged 60 to 75 years in Kermanshah retirement centers. After making arrangements with the officials of 10 retirement centers, despite explaining the reasons and objectives of the research by telephone, only the retirees of six centers accepted the invitation to the interview and 14 people refused to participate in the study due to the coronavirus outbreak. In order to achieve the most diverse views and participants, purposive sampling was performed with maximum variation in terms of retirement center (Place of employment before retirement) and demographic variables (Age, gender, number of children and level of education). In addition, to reflect a mix of lower, middle and upper socio-economic strata, the participants were recruited from different regions based on income (low-income, middle-income, and high-income areas and access to free public facilities).

### Data collection tool and methodology

A qualitative study by an interested and experienced research team using in-depth semi-structured interviews was undertaken using a directed content analysis approach [24, 25]. The interviews were conducted by the corresponding author, a doctoral student with qualitative research experience between October and November 2020, after obtaining an informed consent from all participants. Data were collected using in-depth semi-structured and face-to-face interviews at various retirement centers in a comfortable and stress-free room. The following interview guides were used based on the dimensions of health literacy including access, reading skills, comprehension, evaluation, decision making, and use of information.

1. From what sources and how information about physical activity for older adults can be obtained?
2. What is your opinion about reading the contents and recommendations about physical activity that are published in magazines, booklets, educational and promotional brochures, etc.? And what factors affect your reading skills?
3. What is your perception of the content and recommendations for physical activity, and what factors affect your understanding of the content?
4. What is your opinion about the benefits of physical activity for older adults and the problems caused by not doing it?
5. How can the validity and accuracy of information received from various sources in the field of physical activity for older adults be evaluated, and what are the related factors?
6. What factors affect the decision-making and regular physical activity in older adults?
7. How can we use physical activity information and recommendations from different sources?

Of course, these seven questions were the main questions. After asking each main question, probe or following questions were used to achieve the details and more explanation about answers. There were two facilitators, an interviewer and a trained colleague, a Master’s in health education with qualitative research experience who wrote down all the participants’ answers word by word. To complete the content, the interviews were digitally recorded with the permission of participants. In order to observe the ethical principles, the researchers explained the research objectives and reason for using audio recording. They also stressed that all information obtained from the study was confidential and would be used solely for the purpose of the study, and that the participants had the right to leave the study at any time. At the beginning of each interview, the participant would be asked to introduce themselves and then a number of general questions would be asked about their demographic status. Then the exploration of their views, opinions, and perceptions would be continued. The interview would be continued to make sure that the interviewer understood the issues and concepts raised. The interview process continued until the saturation point was reached, meaning that no new information and concepts that needed a new code was obtained, and the continuation of the interview would yield no new data [24]. Totally, 25 interviews were conducted. Each interview lasted between 25 and 60 minutes, depending on the level of response of the participants.

### Data analysis

Content analysis approach was used to analyze the data [25]. Analysis was conducted manually by the first author (PhD), commencing with the transcripts being read several times to grasp the initial meaning. The recorded content would be written verbatim on paper after listening to it for several times, then it would be matched with the interview manuscripts to complete the incomplete items. The transcripts would be analyzed by two researchers (correspondence and second authors, (PhD)) independently using an open coding system. In this way, the transcripts would be converted into meaning units and after summarizing, it would be converted into codes. At this step, in order to achieve a common procedure in data coding, the transcripts encoded by two people would be controlled by two other members of the research team (third and fourth authors, (PhD)). Discrepancies in the rules regarding coding or classification of codes were resolved through discussions between the research team. Different codes were placed in subcategories based on similarities. Subcategories also formed categories based on their relationship with each other or dimensions of health literacy. Eventually, these categories formed the main theme of health literacy in the field of physical activity of older adults. In qualitative studies, the concept of trustworthiness is introduced as a criterion for substituting validity and reliability, which has four elements of Credibility, Transferability, Confirmability, and Dependability [26]. According to the prior agreement to increase the credibility and acceptability of the data, we called people for further explanation and disambiguation in cases where the information obtained from the interviews were ambiguous.

The study was conducted in 37 days through communication and interaction with the participants to gain a proper and deep understanding of the participants. The participants were also asked to review and verify the codes, so that the extracted codes were returned to the participants before being placed in the subcategories. Transferability refers to concepts such as usability and appropriateness [27], which was ensured by selecting participants from different socio-economic classes and diverse demographic spectrums. In addition, by providing accurate and purposeful explanations of the research process and actions taken in the study path, others can follow the research path. To make sure of confirmability, the manuscripts and research findings were given to another researcher for checking the findings [28]. To ensure confirmability of the data, the review method was used by experts, that means two people who had sufficient experience in qualitative research, reviewed the manuscripts and different aspects of the research to reach similar results. Finally, dependability, which is equivalent to reliability in quantitative research, can be achieved through an audit process, the criterion of which is to check the accuracy in relation to the stability of qualitative research findings.

## Results

In the present study, 11 women aged 60–70 years and 14 men aged 60–73 years participated. The average age of participants was 64.24 years and all of them were married. Other attributes of the participants are presented in Table 1.

**Table 1:** Demographic characteristics of study participants (number = 25)

Variable		Female	Male	
Age (years)	Mean ± SD	3.19 ± 61.72	5.25 ± 66.21	4.93 ± 64.24
Age range (years)		70–60	73–60	73–60
Number of children	Mean ± SD	0.92 ± 2.63	1.45 ± 3.42	1.28 ± 3.08
level of education	High school	2	9	11
	Associates’ degree	6	2	8
	Bachelors’ degree	3	3	6
Place of employment before retirement	Education	6	5	11
	Medical Sciences	3	4	7
	Telecommunications	–	3	3
	The environment organization	–	1	1
	Regional Water	2	–	2
	Sports and Youth	–	1	1

Health literacy in physical activity was considered as the main theme and its dimensions formed the categories. By analyzing the manuscripts obtained from the interviews, 59 initial codes were extracted, which were reduced to 32 main codes after careful assessment. The main codes were categorized into 13 subcategories, then the subcategories formed five categories or dimensions of health literacy as the main theme (Table 2). The main codes and subcategories were derived from the data, while the categories or dimensions and main theme were pre- identified. The subcategories or the same factors related to health literacy of older adults in physical activity obtained from the analysis were placed as follows in the categories or dimensions of health literacy.

**Table 2:** Themes categories, subcategories, codes and meaning units

Theme	Category	Subcategory	Code	Meaning units
**Older adults’ health literacy in the field of physical activity**	**Access**	**Facilitators**	**Support of relevant organizations and departments**	Brochures should be prepared with the help of physical education experts, with which older adults can do sports at home/ There should be extensive publicity on television and in the press / The main source of information for exercising is television, but it is better to put free magazines and brochures in shops or distribute them at home at least once a week.
			**Cyberspace and social channels**	Older adults’ participation in meetings and in-person meetings or on virtual channels where sports veterans share their experiences, provide access to information and encourages sports/ I personally get sports content and advice from the internet and we exercise with my family.
			**Support by family and others**	We need to get physical activity information from retirees and older adults who are experienced and currently active, who know what exercise is right for older adults and give us the right information/ Family members, especially young people, can get the right information about sports and give it to us.
			**Positive inner motivations and habituation to sports**	People who have been interested in sport during their young ages tend to follow sport content and information during their old ages/ Older adults should feel the need to acquire information about physical activity and exercise.
		**Barriers**	**Economic and social problems**	Economic problems in society are one of the barriers to accessing information for physical activity/ Older adults are more concerned with family and child problems and less concerned with learning about physical activity.
			**Insufficient attention of the pertinent organizations to sports**	When an employee retires, there is no plan for their physical activity and they are discounted from the society/ Older adults’ sports are not a priority for the pertinent organizations/ There should be centers and clubs where older adults can obtain specialized information for physical activity.
			**Poor belief and attitude towards the benefits of physical activity**	Older adults who do not understand the benefits and importance of exercise have no desire to pursue resources and engage in physical activity/ If there is no previous interest and positive attitude towards sports in older adults, they would not follow the issues related to sports.
	**Reading skills**	**Personal attributes**	**Reading power**	A sports booklet is good for well-educated older adults who understand and practice/ I like writing material, but it is difficult for us, with poor eyesight.
			**Interest**	It is difficult to read physical activity texts, but I read these topics because I am interested/ For those who like to read, this is good, but many older adults who are not interested, tend to become bored easily.
			**Activity habit**	Individuals who are physically active tend to read materials about physical activity/ Older adults must have the perseverance to read the materials about physical activity/ If an old person has experience and habit of physical activity, they will follow the content.
		**Quality of writing and content**	**Writing structure**	Content should make older adults thirsty for reading and have a fascinating representation and images that create enthusiasm in older adults / Appropriate, scientific, simple, concise and attractive information will be useful.
			**According to need**	The content should be specialized and tailored to the needs of older adults and complete/ The movements shown in the books should take into account the physical and mental condition of older adults and be safe for their age.
		**Distribution source characteristics**	**Source validity, frequency and continuity of publication**	Content should be published periodically rather than temporary. The publication of print media should be continuous, at large-scale and from authoritative and official sources/ References should be provided by expert and well-known people.
			**Easy and free access**	It will definitely be useful if the print media is constantly distributed in large quantities and distributed free of charge/ It is better if there are places where older adults can easily and freely access the contents.
	**Comprehension**	**Facilitators**	**Paying attention to the consequences of having and not having an activity**	If I do not exercise for a week, my whole body aches and life becomes difficult / With exercise, I do not suffer from depression, loneliness, frustration, low self-esteem, muscle loss, weakness, hypertension, heart disease, diabetes, hyperlipidemia and early death. By doing physical activities, I am always happy, less in need of others, feel valued and useful, and have good relationships with family and others.
			**Effective training**	To make older adults person becomes interested and pursues sports, sports content and tips should be fun and entertaining so that older adults can enjoy the practice/The recommender should present the content with good morals, cheerful spirit, sweet and pleasant expression, and sometimes even with humorous language.
			**Have a positive experience**	Based on our ability, we will practice sports recommendations at least once and if we have a good experience, we will continue.
			**Organizational support**	Individuals are abandoned after retirement, organizations must provide appropriate facilities and programs for recreation, sports, pilgrimage, and tourism, and draw their attention to sports.
		**Barriers**	**Individual problems**	Isolation, depression, illness, and stress can prevent you from paying attention and understanding physical activity.
			**Socio-economic barriers**	Most retirees have economic problems such as education and employment of their children, they are not comfortable with these problems and cannot think about sports and physical activity.
	**Evaluation**	**Internal evaluation**	**Background and experience of older adults**	The best source for assessing the accuracy of the recommendations is older adults themselves, If following an advice makes older adults feel fresh and has a good effect on health, it will be considered valid/ The activity and movement that is recommended to older adults should be appropriate for their condition and should be very simple and light so that they can continue the practice without harming themselves.
		**External evaluation**	**Confirmation of reliable and experienced people**	I try to get information from university professors and people around me who are reliable and experienced in sports/ Information from coaches and sports veterans is considered as reliable.
			**Obtain information from reputable sources**	I use websites of which I know the coaches from TV shows and books whose authors are experts on sports / To evaluate sports content or recommendations, the first step is to identify and search the source and verify it through retirement and sports organizations.
	**Decision-making and application of information**	**Individual factors**	**Believe and understand the benefits**	Older adults’ own beliefs and perceptions of the importance of exercise are involved in making decisions and engaging in physical activity/ Thinking about dealing with physical problems and illnesses in old age and the inability to do one’s work makes one to decide to do physical activity/ By doing physical activity, older adults feel more relaxed and can rely on themselves as much as they can, and avoid becoming a burden on their families.
			**Positive experience**	Older adults should follow the recommendations and after receiving a good result, they will definitely decide to continue exercising.
		**Interpersonal factors**	**Support others**	Family and friends can encourage older adults to make decisions and exercise by telling them the benefits of exercise
			**Consultation with experts**	After consultation with coaches and doctors, we should start with very light movements and beginners’ steps/ Motivation is created by referring to those who have expertise and experience in sports and receive information from them about the benefits of sports.
			**Interact with active patterns and groups**	We can go to cultural and sports centers with family, neighbors, and friends and participate in group sports or have a daily walk and exercise program in the park/ Associating and interacting with older athletes create the desire and will to do sports.
		**Organizational factors**	**Organizational planning and advertising**	Advertising in various media and planning by the pertinent organizations to keep retirees active is important so that people, after retirement, can have an active package and program to have an active way of life.
			**Organizational facilities**	Organizations should provide facilities for the physical activity for older adults by planning and cooperating with retirement centers.
		**Socio-economic factors**	**Economic situation and family problems**	One of the most influential factors is the economic and livelihood problems that have plagued many retirees as these problems deprive older adults of the peace of mind and they should think about livelihood instead of sports.
			**Status of sports venues and spaces in the community**	Creating and developing sports facilities and spaces in communities play an important role in motivating, making decisions, and performing physical activity by older adults.

Bold entries are significant

### Access category

Access category includes two subcategories of facilitators and barriers to accessing information. Participants mentioned several sources and related factors for accessing physical activity and exercise information. These sources and factors included various organizations (physical education, radio and television, welfare, pension fund and health centers, and bases), cyberspace, books, magazines, support and advice of others (family members, colleagues and friends, older adults athletes, sports coaches in clubs and parks, doctors), interest, positive perceptions, and exercise habits.

*‘There should be extensive publicity on television and in the press. Posters and leaflets should be placed at health centers and clinics’* (P21).

Participants also believed that there were problems that prevented older people from accessing information and physical activity recommendations. Deterrents included economic and social problems, inadequate attention by various pertinent organizations and departments to sports, lack of facilities, and poor belief in the benefits of physical activity.

*‘I think the economic problems are one of the barriers to accessing physical activity information. Because of these problems, the publication and access to sports magazines are also very limited’* (P24).

### Reading skills category

Although, some older adults were reluctant to read physical activity content and recommendations, most of the participants found the factors in encouraging older adults to engage in physical activity through reading the print media. These factors included interest in the subject, reading ability and having a history of physical activity, quality of writing and text content (simplicity, clarity, attractiveness of writing with images, completeness, and scientific and appropriate to the needs according to specific conditions and diseases of old age), source validity, frequency and continuity of publication, and easy and free access.

*‘It will definitely be useful if the print media is continuously published and distributed free of charge from reputable and official sources. Scientific information should be provided in a simple, attractive, and appropriate language based on the needs of older adults’* (P4).

*‘People tend to read the articles and follows the sports news, when they are interested in sports or have a history and habit of sports,* (P6).

### Comprehension category

Most of the participants explained the importance and benefits of being physically active and the problems and harms of being inactive. They mentioned facilitators and barriers of understanding physical activity content. Facilitators included attention to the consequences of having or not having physical activity, effective training, organizational support, and having a positive experience. The barriers included personal, economic, and social problems.

*‘Sitting at home and being inactive makes the life hard so that it is difficult for us to do daily activities and interact with family and others’* (P1).

The participants mentioned several educational factors in having a better understanding of the concepts. These factors included the quality and attractiveness of the instructor’s speech, fun and entertaining content, providing an active role model, providing reliable and correct recommendations and content, observing movements objectively, and transfer of sports experiences during the gatherings of older adults.

*‘Seeing the movements in practice, for example, showing the correct movements of the hands, feet and neck on TV is much better and older people can learn to do the movements perfectly’* (P2).

### Evaluation category

The effective factors expressed in the evaluation category were placed in sub-categories of internal evaluation and external evaluation. In internal evaluation, the validity of information is measured based on the background and experience of older adults in physical activity and sports, while in external evaluation, the validity and accuracy of information is assessed based on the approval by reliable and experienced people and receiving content from reliable sources.

*‘I try to get information from radio and television, university professors, coaches, and people around me who are reliable and experienced in sports’* (P7).

*‘The best source for assessing the correctness of the recommendations is older adults themselves. If the advice makes older adults fresh and has a good effect on their health, it is considered valid’* (P5).

### Decision-making category and application of information

Decision-making category and application of information was influenced by individual, interpersonal, organizational, and environmental factors. Individual factors included believing in and understanding the benefits of physical activity such as self-reliance, not being overwhelmed, enjoying life, the positive experience of older adults through planning, and devoting time to daily physical activity. Interpersonal factors included supporting and encouraging others, consultation with coaches, experts, and veterans, interacting with role models, and joining active groups. Organizational factors included planning, advertising, and developing physical activity facilities for retirees. Moreover, socio-economic factors included the economic status of the family and the places and spaces and facilities available for physical activity.

*‘If the benefits of exercise are expressed by those around and family, older adults are encouraged to make decisions and exercise, Family support, for older adults, leads to their life satisfaction and happiness. Family is an important factor in making decisions’* (P18).

Most of the participants emphasized on socio-economic problems as one of the barriers to physical activity; however, a few did not consider these problems as obstacles to activity.

*‘Life condition does not affect exercising. Exercise is not expensive and has nothing to do with economic or social problems. You can exercise at home or walk every day’* (P16).

After presenting the findings to the enthusiastic participants, they provided feedbacks to the study by proposing to apply the results in the community.

## Discussion

The findings of this study were analyzed based on the five dimensions of health literacy in the field of physical activity of older adults, including access, reading skills, comprehension, evaluation, decision making, and application of information, each of which represented by a category. Almost all participants preferred TV programs, watching live sports and virtual channels over print media. Consistent with this finding, Shabani et al. concluded that morning exercise shows on radio and television was an effective factor in promoting physical activity [29]. Most of the participants believed that, in addition to being aware of the benefits of physical activity, conscious awareness of physical and psychological problems that are the result of inactivity can be effective and motivating in their understanding of the importance of physical activity. Therefore, attention to the negative consequences of inactivity in older adults was considered as a facilitator. Hassan Pour and Abbasi noted that raising the knowledge and awareness in older adults about the benefits of sports was one of the effective factors in the participation of older adults in sports activities (Hasan Pour Azghadi and Abbasi 2006). The participants mentioned facilitators such as effective training to better understand physical activity recommendations. These findings are consistent with the results by Tehrani et al. about educational-communicative factors affecting the health literacy [30].

From the participants’ point of view, understanding the benefits of physical activity, having a positive activity experience, support by others, consultation with coaches and interacting with active role models and groups, advertising and organizational facilities, socio-economic situation and community facilities were involved in decision making and physical activity. These findings are similar to the results of a qualitative research by Sarani et al., which was in the form of individual and organizational themes and the factors affecting the participation of Iranian older adults in sports activities [31]. In this study, social support was a motivating factor for physical activity, life satisfaction and happiness, which is consistent with the results of a study by Moeini et al. who showed that educational intervention based on social support theory can play a pivotal role in promoting mental health, life satisfaction, and happiness of older adults [32].

In a systematic review, Smith et al. showed that people with social support, especially from family members, do more physical activity in their spare time (Smith et al. 2017). These results are similar to the views of the participants in the present study on the decision-making and application of information. The participants believed that older adults, based on their ability and positive experiences of physical activity would evaluate the information of physical activity and decide to take action. This finding is consistent with Geboers et al., who considered self-efficacy as a mediation between health literacy and execution of physical activity instructions [15]. Moreover, according to Notthoff et al., health problems and psychological factors such as mental health, motivation, self-efficacy, and health perception are consistently associated with high levels of physical activity in older adults [33].

Findings of the present study on decision making and application of information are consistent with the results by Nadri and Safania, who explained the factors in motivation or inhibition of physical activity of older adults. They introduced subcategories intrapersonal, interpersonal, and environmental factors [34]. However, some of the participants mentioned problems, mental and physical conditions, and illness in old age as barriers to perceiving the value of physical activity. However, none of them mentioned the physical problems and diseases of old age as an obstacle to making decisions and applying physical activity recommendations. In addition, they believed that ageing effects depends on one’s ability and circumstances. This means that after going through the stages of understanding and evaluating information, physical problems and illnesses are not obstacles to making decisions and applying physical activity information.

Overall, based on of this study, organizational factors such as support, advertising, and organizational facilities had a role in all aspects of health literacy. Social and economic problems were common barriers for older adults with dimensions namely access, understanding, decision-making, and use of physical activity information. Sadrollahi et al. concluded that the reduction of social and environmental problems and providing a suitable place were the determining and effective factors in increasing physical activity in older adults [35]. Jovic Vranes et al. supported the effect of environmental and cultural factors on the level of health literacy [36].

Nazari (2018) examined the future of older adults sports using a matrix of interactions [37]; the most important factors affecting the future of older adults sports in Iran were paying attention to the position of older adults, creating local organization, allocating funds, modeling other countries, designing long-term plans, and creating process-oriented process strategies. These findings are consistent with the results about the organizational factors in decision-making and application of information in the present study. Sarani et al. listed the strategies for promoting the health of older adults through sports activities (Sarani et al. 2018a); including, establishment of older adults sports organization, development of strategic and operational plans for sports development, establishment of rules for older adults sports, development of sports places and spaces for older adults, provision of specialized human resources, establishment of a research and development unit for elderly sports, promotional works, service quality improvement, development of sports culture, and funding. These findings are consistent with the present study on the role of organizational factors in decision making and application of information in the field of physical activity.

## Limitations

One of the limitations of the present study was the time-consuming process of obtaining licenses and making the required arrangements with the relevant officials. Additionally, the coincidence of the Covid-19 pandemic and interviewing process was another limitation of this study because it made it very difficult to access people. Although we visited all 10 retirement centers to conduct the interviews after making arrangements with the officials, the research team managed to interview the retirees only in six centers. Another limitation was the target group i.e., retirees do not fully reflect the whole elderly community in a country, thus the results of this study cannot be generalized to the whole elderly community in the country. There is no doubt that designing and implementing more extensive studies over a wider range of the elderly class in different areas of the country provides a more thorough and comprehensive understanding of the beliefs and factors concerning the health literacy about physical activities of the elderly that can contribute to formulation and implementation of the plans and policies applied to promote the health status of the elderly.

## Conclusion

This study provided a comprehensive understanding of beliefs, opinions and factors related to older adults’ health literacy about physical activity. According to these findings, physical problems and diseases do not prevent the decision-making about physical activity and application of physical activity information in older adults who have a high understanding and proper evaluation of physical activity recommendations. Support, advertising, and organizational facilities are related to all dimensions of older adults’ health literacy about physical activity, while socio-economic factors are related to the dimensions of access, understanding, decision-making, and application of information.

The important and practical point in conducting this research is that future researchers can make use of its results to develop some specific tools to assess the health literacy of the elderly in the field of physical activities. After the assessment of the health literacy and physical activities of the elderly as a background and fundamental variable, they can develop and implement some interventions considering the relevant factors concerning the dimensions of the study in order to promote physical activities. In addition, the results of this study can be used by organizations and institutions that are responsible for policy-making, decision-making, and implementing physical activity promotion programs to improve the health in older adults.

## Data Availability

The datasets used and/or analysed during the current study are available from the corresponding author on reasonable request.
